# Cross-shelf and vertical structure of pelagic amphipods (Crustacea) related to hydro-meteorological conditions in the neritic zone, southern Gulf of Mexico

**DOI:** 10.1371/journal.pone.0336930

**Published:** 2025-12-04

**Authors:** Marco Violante-Huerta, Laura Sanvicente-Añorve, Miguel Alatorre-Mendieta, Edlin Guerra-Castro

**Affiliations:** 1 Laboratorio de Ecología de Sistemas Pelágicos, Instituto de Ciencias del Mar y Limnología, Universidad Nacional Autónoma de México, Mexico City, Mexico; 2 Laboratorio de Oceanografía Física, Instituto de Ciencias del Mar y Limnología, Universidad Nacional Autónoma de México, Mexico City, Mexico; 3 Laboratorio de Ecología Marina, Escuela Nacional de Estudios Superiores-Unidad Mérida, Universidad Nacional Autónoma de México, Mérida, Yucatán, Mexico; L3 Scientific Solutions, GERMANY

## Abstract

The environmental drivers that effect the pelagic amphipod communities in the neritic province have received little attention worldwide. This study analyzed the influence of fluvial discharges and wind regime on the pelagic amphipods in neritic waters of the southern Gulf of Mexico during two contrasting seasons: May (*dry*, lower discharges and wind speeds) and November (*nortes*, higher discharges and wind speeds). Zooplankton samples were collected at five depth levels of the water column (0–6, 6–12, 12–18, 45–55, and 95–105 m) using a stratified opening-closing net system. Environmental gradients were assessed by defining three horizontal assemblages (‘coastal’, ‘neritic’, ‘slope’) and two vertical assemblages (‘surface’: 0–18 m; ‘deep’: 45–105 m). Seasonally, the greater volume of nutrient-rich discharges during *nortes* resulted in a higher amphipod density. Spatially, low salinity waters off river mouths corresponded to the lowest amphipod density and diversity. The horizontal and vertical assemblages showed significant differences during the *dry* (ANOSIM test, *p* < 0.05); during *nortes*, the structure of horizontal and vertical assemblages showed a lower heterogeneity, probably due to wind strength. Differences within the assemblages defined in the horizontal and vertical planes were only evident when *Lestrigonus bengalensis* was considered in the analysis; however, without this species, the amphipod community showed more homogeneity across the two seasons. *Anchylomera blossevillei, Tetrathyrus forcipatus,* and the juveniles of Eupronoidae were ecologically relevant in the characterization of the assemblages due to their specific environmental requirements. Diversity, evaluated using the completeness method, increased from the coast to the ocean in both seasons; vertically, diversity was highest in the ‘deep’ assemblage during the *dry*, but no differences were observed between assemblages in *nortes*. The greater similarity of the assemblages during *nortes* was associated with a higher homogeneity in hydrological conditions due to the strong winds that dominate this season. These findings contribute to a deeper ecological understanding of the effects of hydro-meteorological conditions on the structure of pelagic amphipod communities.

## Introduction

Pelagic amphipods generally have a cosmopolitan or circumtropical distribution [1,2]. Most species have a clear preference for inhabiting the oceanic province, where the highest diversity was recorded [1]. Instead, the highest abundance of amphipods is found in the neritic province [3]. Among the pelagic amphipods, the suborder Hyperiidea stands out for its dominance and species diversity, since it is an exclusively holoplanktonic group, of which 292 species have currently been recognized [1,2,4,5]. Furthermore, some species from suborders Amphilochidea and Senticaudata are found in the holoplankton of epi-, meso-, and abyssopelagic waters [6–9].

Globally, the ecology of pelagic amphipods has been studied mainly in the oceanic province, and their community structure has been related to temperature and productivity gradients, as a result of diverse mesoscale and coarse-scale oceanographic processes [4,9–12]. However, in the neritic province, the pelagic amphipod community has been poorly studied. Some studies highlight the high abundance of this zooplankton group in neritic waters, but do not provide further ecological data on the community structure [1,3,13]. Only a few studies address the influence of the hydrological processes on the pelagic amphipod community structure in the horizontal plane [14–17]. Among the oceanographic processes that influence the distribution of zooplankton in the neritic zone are the marine currents [18–21], the thermocline depth [22], upwelling processes [23], and continental water discharges [21,24,25] that vary throughout the annual cycle.

Mexico, which has an extensive coastline, has many rivers and lagoons that discharge freshwater, sediments, and organic and inorganic matter into the marine system. Particularly, the greatest hydrological contributions to the Gulf of Mexico are the Mississippi River to the north and the Grijalva-Usumacinta River to the south [26–27]. In the southern Gulf, previous studies have emphasized the importance of the continental water discharges over the distribution and structure of some zooplankton organisms, such as holoplanktonic mollusks [28–31], fish larvae [32], and siphonophores [33]. An additional factor to consider in this region is the seasonal variation in wind patterns, with stronger winds occurring from October to March during the *nortes* season [34–36], whose effect on zooplankton is scarcely explored. Currently, the implications of the wind regime and the continental discharges on the pelagic amphipod community in the region are unknown, so two main questions need to be answered to better understand the mechanisms that structure this community in the neritic province of the Gulf:

1) what is the effect of the continental water discharges on the amphipod community?
2) how does the stronger wind regime that occurs during the *nortes* influence the community structure in both the horizontal and the vertical planes?

This study explores the horizontal and vertical variations in the structure of the pelagic amphipod community in the neritic waters of the southern Gulf of Mexico, during two contrasting seasons in terms of rain and wind regimes: May (*dry* season) and November (*nortes* season).

## Materials and methods

### Seasonality

In the southern Gulf of Mexico, three main meteorological periods have been recognized considering rainfall intensity and wind regime: *dry*, *rainy,* and *nortes* seasons. The *dry*, lasting from February to May, is characterized by moderate wind speed (4–6 m/s), and the lowest pluviosity in the year (< 300 mm). The *rainy*, from June to September, the wind speed is similar to the *dry* period, but the rainfall is the highest throughout the year (800–1200 mm). Finally, the *nortes*, from October to March, is characterized by sporadic rainfalls and strong cold winds coming from the north that reach speeds of up to 8 m/s, which diminish the surface water temperature [34–36]. It is worth noting that interannual variability in the seasons’ periods exists [37].

Continental water discharges gradually increase from June to September-October, with values ranging from 1,600–3,900 m^3^/s; after which they decrease [38]. This means that the highest discharges occur from the end of the *rainy* to the beginning of *nortes* one, and the lowest discharges happen at the end of *nortes* and the entire *dry*.

### Sampling and laboratory work

**Fig 1:**
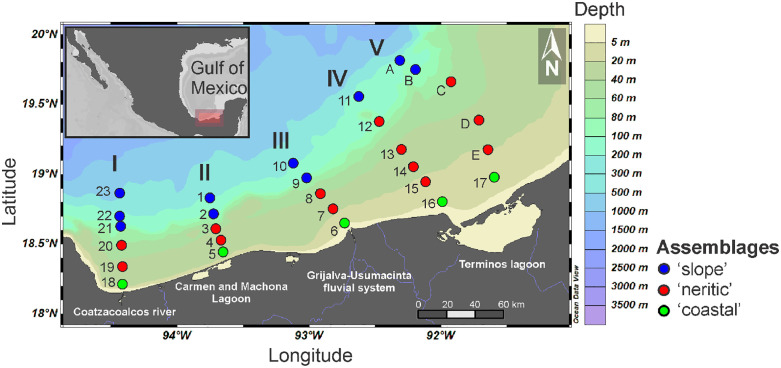
Sampling sites arranged by transects (I-V) and location of the horizontal assemblages defined in the neritic province of the southern Gulf of Mexico. Republished from Ocean Data View under a CC BY license, with permission from Prof. Dr. Reiner Schlitzer (rights holder), original copyright 2025.

Sampling was performed aboard the R/V “Justo Sierra” during the Project entitled “Monitoreo de Pre-reclutas de Especies Estuarino-Dependientes” (MOPEED), carried out in neritic waters of the southern Gulf of Mexico in 1995. Two oceanographic campaigns were performed during two contrasting seasons: *dry* in May and *nortes* in November. In each season, 28 sampling stations were organized into five transects perpendicular to the coastline in front of the main continental water discharges of the region: Coatzacoalcos River (I), Carmen and Machona lagoon system (II), Grijalva-Usumacinta fluvial system (III) and the Terminos lagoon (IV and V). Sampling stations were ploted in horizontal maps (Fig 1) using the Ocean Data View (ODV 5.8.3.) software [39]. Temperature and salinity were recorded before the zooplankton sampling, using a CTD sonde (Sea-Bird SBE 9) deployed from the surface (0 m) up to 250 m depending on the bottom depth. This sonde uses a pomp and a TC (temperature-conductivity) duct to ensure a constant flow, allowing both sensors to measure the same water mass and reduce salinity spikes. Afterward, zooplankton was collected at one or up to five levels of the water column, from the surface up to 105 m depth: level 1 (0–6 m), level 2 (6–12 m), level 3 (12–18 m), level 4 (45–55 m), and level 5 (95–105 m); the number of sampled levels depended on the bathymetry. Circular double oblique tows were conducted using a stratified opening-closing net system (75 cm mouth, 500 µm mesh, 2 m long) during 25 minutes at 3 knots. Additionally, an inclinometer was employed to measure the sampling depth using trigonometry. In total, 187 zooplankton samples (94 in *dry* and 93 in *nortes*) were collected because not all sampling stations were suitable for collecting samples at all five levels due to the shallower bottom depths. The biological material was fixed in a 4% formalin solution and preserved in 70% ethanol. These samples form part of a project initially conceived in the 1990s to evaluate the influence of continental water discharges on fish and shrimp larvae in the upper layer of neritic waters of the southern Gulf; the deep levels were taken as a complement, not as a priority.

In the laboratory, zooplankton biomass was measured using the displacement volume method [40], and data were expressed as mL/1,000 m^3^. Then, all amphipods were sorted from the samples and identified according to specialized literature [1,2,8,41–44]. Most of the juvenile organisms were not suitable for identification due to their incomplete development and high similarity to other species, which could lead to errors in the identification of taxa. However, they were considered in the formal analysis (e.g., family Eupronoidae, genus *Primno*). The amphipod abundance data were standardized and expressed as ind/1,000 m^3^ (available at https://doi.org/10.5281/zenodo.17298974).

### Data analysis

#### Hydrology.

Temperature and salinity data recorded from CTD were used to estimate the mean integrated value for each sampling level using the following formula:


$$Mx=\frac{\text{1}}{\left(Z_{f}-Z_{i}\right)}\int_{Z_{i}}^{Z_{f}}x(z)dz$$

Where *Mx* is the integrated mean value of *x*, *x* is the parameter (temperature or salinity), *Z*_*i*_ is the initial sampling depth, *Z*_*f*_ is the final sampling depth, and Z is the depth of the integrated variable.

Mean integrated values were used to plot the horizontal distribution of the surface temperature and salinity to identify the influence of the water discharges and to perform the multivariate analyses. Additionally, temperature and salinity data were plotted in vertical profiles along the five transects to observe the distribution of the parameters with depth using the SURFER v15 software.

#### Biological data.

To identify the environmental gradient generated by the influence of continental water discharges over the structure of the amphipod community, we defined three horizontal assemblages (named ‘coastal’, ‘neritic’, and ‘slope’) and two assemblages in the vertical plane (‘surface’ and ‘deep’).

In the first step of data analysis, which explored the horizontal structure, the ‘coastal’ assemblage was composed of the stations located closer to the major continental water discharges; the ‘neritic’ assemblage comprised stations on the continental shelf; and the ‘slope’ consisted of stations located over the limit of the shelf (Fig 1). Matrices crossing density of amphipod species and sampling stations of each sampling season were transformed by applying a square root to reduce the bias of the dominant species and to meet the normality assumption. Then, to determine which variables (temperature, salinity, zooplankton biomass, amphipod density, and distance to the shore) were associated with each of the assemblages, a Principal Component Analysis (PCA) was performed on the transformed data; as a complement, the amphipod density (ind/1,000 m^3^) was represented as bubbles at each data point (sampling station). Afterward, an ANOSIM hypothesis test (9999 permutations) was applied to a station’s Bray-Curtis similarity matrix to prove the differences among the three assemblages. The ANOSIM (Analysis of Similarities) test considers the rank of the data to assess differences among groups. The proof uses the rank permutations to calculate the statistical significance, with a resulting *R* statistic indicating the degree of similarity: *R* values near 1 indicate differences between data sets, while values close to 0 indicate resemblance between groups. This non-parametric method has no strict assumptions about the underlying data distribution, such as normality or homoscedasticity [45]. Furthermore, a SIMPER analysis was applied to determine the taxa with the greatest contribution to groups’ discrimination. These analyses were made using the PRIMER v7 software [46]. These multivariate procedures were replicated without the dominant species (*Lestrigonus bengalensis*) to evaluate the effect of this species on the amphipod community structure.

Horizontal assemblages were also compared in terms of diversity, evaluated through the number of species. In both seasons, species accumulation curves of the alpha diversity were performed with the interpolation-extrapolation of the Hill numbers and the incidence-based estimator Chao2 [47]. Moreover, sample completeness was estimated for each assemblage to infer the representativeness of the sampling effort [47] in both seasons, considering a common sample coverage value of 0.75 to compare the interpolated alpha diversity. “Completeness” represents the probability of obtaining the species already recorded in the next sample. In traditional methods to estimate species richness, all species are treated as equal, but their abundances are completely ignored. By contrast, if individuals are equally considered, in a manner that each species is weighted by its abundance, it is possible to estimate the coverage or completeness of a sample [48]. Then, the method allows for comparing species richness of several communities based on samples of equal completeness instead of equal size. The method yields less biased comparisons of species richness between datasets that result from different sampling efforts, allows communities to be ranked according to their true richness, and meets the principle of replicability without loosing any sampling effort, which is essential when discussing diversity [47]. The diversity analysis was performed with the *R* free software and the iNEXT package [49].

In the second step of data analysis, the vertical structure of the community was explored, considering levels 1–3 (0–6, 6–12, and 12–18 m) as the ‘surface’ assemblage, and levels 4–5 (45–55, and 95–105 m) as the ‘deep’ assemblage. In this second approach, the methods used in the horizontal analysis were applied to verify the differences in the community structure in the water column during both seasons. The common sample coverage value used in this second analysis was 0.95.

## Results

### Hydrology

**Fig 2:**
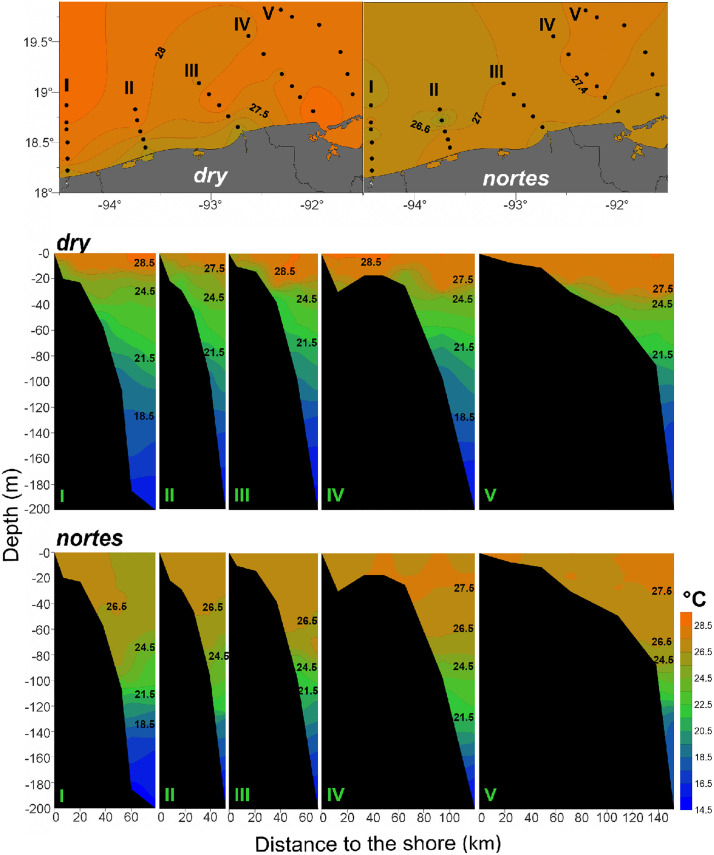
Horizontal and vertical temperature (°C) plots of the neritic province of the southern Gulf of Mexico, during the *dry* and *nortes* 1995. Horizontal representation corresponds to the 0-6 mean integrated value.

Seasonally, the highest temperature values (~28°C), evident in surface waters (0–6 m depth), were recorded during the *dry*; in *nortes*, temperatures were below 28°C. In the horizontal plane, temperatures increased from the coast toward the ocean in both seasons; in the vertical plane, the thickness of the warm water layer (>24.5°C) reaches 40 m in *dry*, and 80 m in *nortes* (Fig 2).

**Fig 3:**
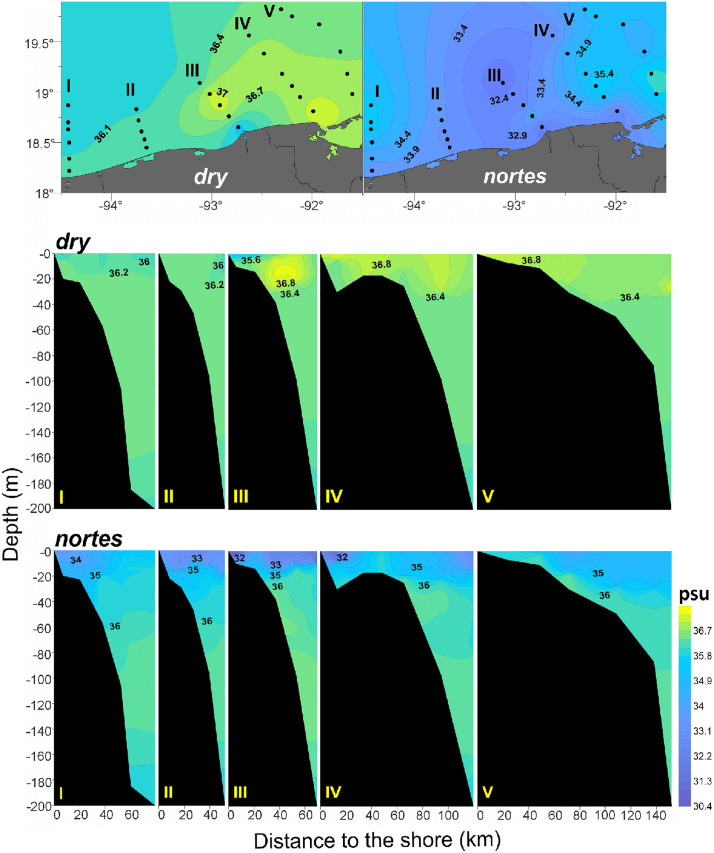
Horizontal and vertical salinity (psu) plots of the neritic province of the southern Gulf of Mexico, during the *dry* and *nortes* 1995. Horizontal representation corresponds to the 0-6 mean integrated value.

In the horizontal plane, surface salinity (0–6 m depth) increases from the coast toward the ocean during *dry,* while in *nortes,* the lowest values (around 32 psu) were observed in the intermediate zone of the study area, indicating the greater effect of the continental water discharges during *nortes*. In the vertical plane, lower salinity (30–32 psu) was recorded between 0 and 40 m depth in the transects III and IV, evidencing the higher continental water discharge during *nortes* (Fig 3).

### Community structure in horizontal and vertical planes

#### Seasonality.

A total of 39,329 pelagic amphipods were sorted and identified across the seasons surveyed. 86 taxa were identified (72 during *dry*, and 74 in *nortes*) and most of them occur in both seasons (S1 Table). The mean (± SD) density of amphipods was slightly higher in *nortes* (1,170.8 ± 2,098.9 ind/1,000 m^3^) than in *dry* (1,059.5 ± 1,538.5 ind/1,000 m^3^). ANOSIM test (*R* = 0.016, *p* = 0.024) confirmed that seasons have significant differences in the density of amphipods.

#### Cross-shelf assemblages.

**Fig 4:**
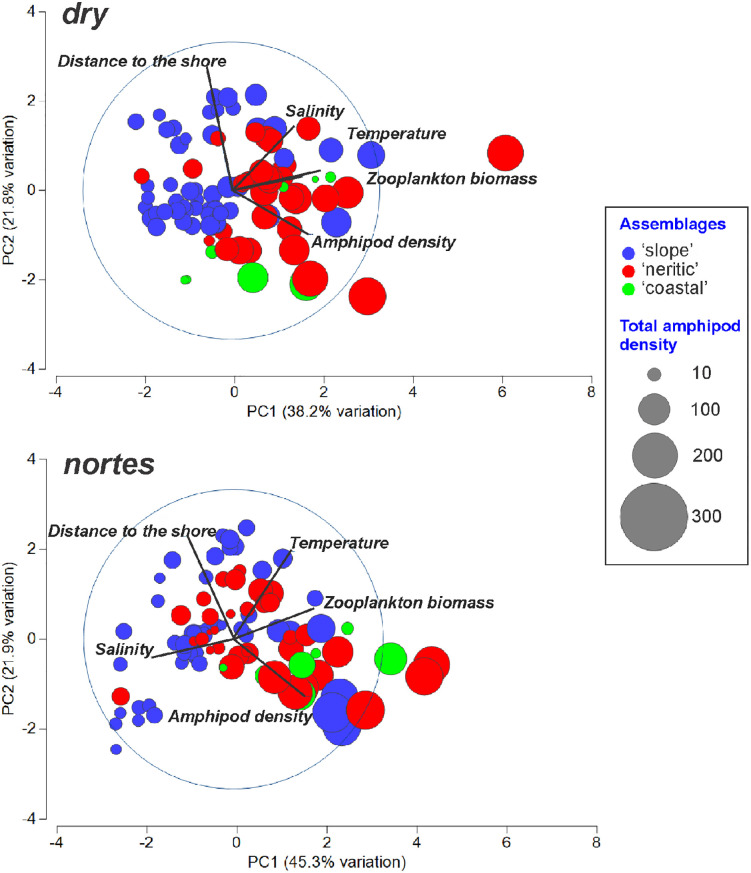
Representation of horizontal assemblages resulting from the Principal Component Analysis (PCA) applied to data obtained during *dry* and *nortes.* Size of bubbles corresponds to the total amphipod density transformed into square root.

During the *dry*, differences in the community structure among assemblages were significant (ANOSIM test, *p* < 0.05). The first two axes of the PCA accounted for 60% of the total variation; on axis 1, the zooplankton biomass was the variable with the highest correlation (Table 1) and was associated with the ‘neritic’ assemblage, characterized by a high amphipod density (Fig 4). During *nortes*, ‘neritic’ and ‘coastal’ assemblages did not show significant differences (ANOSIM test, *p* = 0.563). In the PCA ordination plot, axes I and II accounted for 67.2% of the total variance, and the salinity was the variable with the greatest correlation on axis 1 (Table 1), which showed a negative correlation with the ‘coastal’ and much of the ‘neritic’ assemblage (Fig 4). Results of a supplementary analysis excluding the most abundant species, *L. bengalensis*, indicated that the ‘coastal’ and ‘neritic’ assemblages showed no significant differences regardless of the season (S1 Text: ANOSIM test, *p* > 0.05).

**Table 1:** Correlation between environmental variables and the first two axes of the Principal Component Analysis.

Variable	*Dry*		*Nortes*	
	PC1	PC2	PC1	PC2
Distance to the shore	−0.169	0.841	−0.306	−0.684
Temperature	0.457	0.093	0.377	−0.580
Salinity	0.416	0.426	**−0.538**	0.117
Zooplankton biomass	**0.572**	0.128	0.511	−0.200
Amphipod density	0.512	−0.294	0.463	0.377
Total variance (%)	38.2	21.8	45.3	21.9

**Fig 5:**
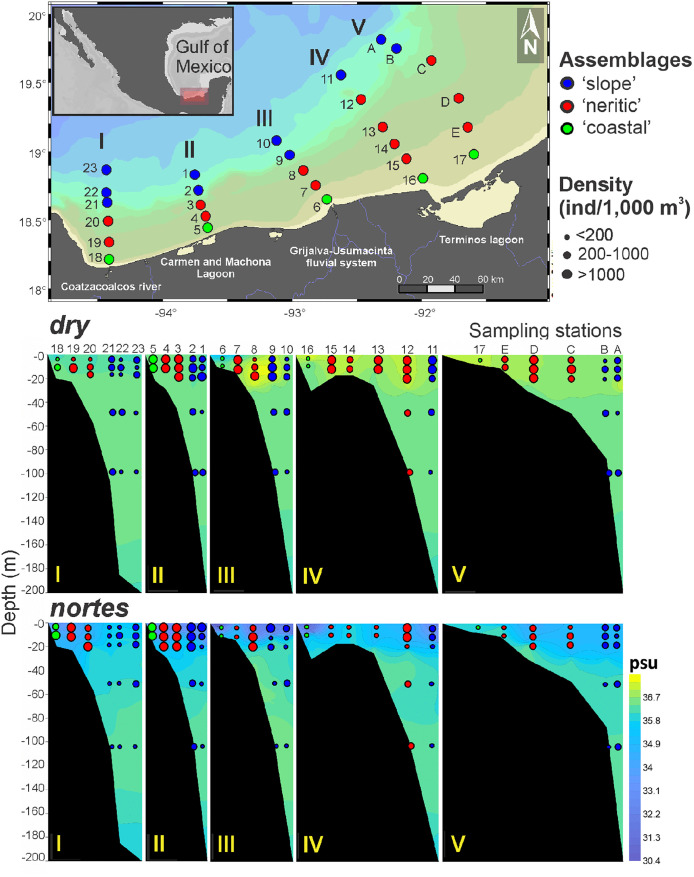
Density (ind/1000m^3^) of pelagic amphipods in the neritic province of the southern Gulf of Mexico during *dry* and *nortes.* Republished from Ocean Data View under a CC BY license, with permission from Prof. Dr. Reiner Schlitzer (rights holder), original copyright 2025.

Overall, the highest density of amphipods was registered in the ‘neritic’ assemblage, while the lowest was found on the ‘slope’ (S1 Table). In particular, the highest density values (~1,000 ind/1,000 m^3^) were found in the transect located in front of the Carmen and Machona lagoon system in both seasons (Fig 5, Transect II). The lowest density values ($\bar{x}$ = 30.7 ind/1,000 m^3^) were recorded in the ‘coastal’ assemblage, particularly in the stations located in front of the Grijalva-Usumacinta system and the Terminos lagoon in both seasons (Fig 5, Transects III to V).

Species composition varied among assemblages, and the highest number of exclusive species occurred on the ‘slope’ assemblage in both seasons (*dry* = 28 species; *nortes* = 27). Among these species, those from the infraorder Physosomata showed the greatest affinity to oceanic conditions, with all species occurring on the ‘slope’ (S1 Table).

*Lestrigonus bengalensis* was the dominant species during both *dry* (78.4% of the total abundance) and *nortes* (86.4%). The species was more abundant in the ‘neritic’ and ‘coastal’ assemblages, and the difference with the ‘slope’ assemblage was more evident during the *dry* (S1 Table). The SIMPER analysis confirmed that *L. bengalensis* recorded the greatest contribution to the assemblages’ discrimination in both seasons. *Tetrathyrus forcipatus*, the juveniles of Eupronoidae, and *Anchylomera blossevillei* were also key players in the assemblages’ differentiation (Table 2), and coincided with the supplementary analysis excluding *L. bengalensis* (Table D in S1 Text).

**Table 2:** Species discriminating the cross-shelf amphipod assemblages according to the SIMPER analysis during *dry* and *nortes.*

*Dry*		*Nortes*	
Taxa	Contribution %	Taxa	Contribution %
	‘slope’ vs ‘neritic’ Av. Diss. = 75.86		‘slope’ vs ‘neritic’ Av. Diss. = 72.90
*L. bengalensis*	29.67	*L. bengalensis*	29.91
*A. blossevillei*	4.98	*T. forcipatus*	5.69
Eupronoidae juv.	4.96	Eupronoidae juv.	5.60
*E. intermedia*	3.63	*B. crusculum*	4.19
	‘slope’ vs ‘coastal’ Av. Diss. = 82.50		‘slope’ vs ‘coastal’ Av. Diss. = 73.56
*L. bengalensis*	19.24	*L. bengalensis*	28.49
Eupronoidae juv.	5.92	Eupronoidae juv.	6.33
*A. blossevillei*	5.63	*T. forcipatus*	5.46
*T. forcipatus*	4.10	*B. crusculum*	4.72
	‘neritic’ vs ‘coastal’ Av. Diss. = 69.89		‘neritic’ vs ‘coastal’ Av. Diss. = 60.85
*L. bengalensis*	52.92	*L. bengalensis*	45.41
*T. forcipatus*	5.80	*T. forcipatus*	8.21
*A. blossevillei*	4.11	*B. crusculum*	6.01
*C. scleroticus*	3.60	Eupronoidae juv.	5.88

**Fig 6:**
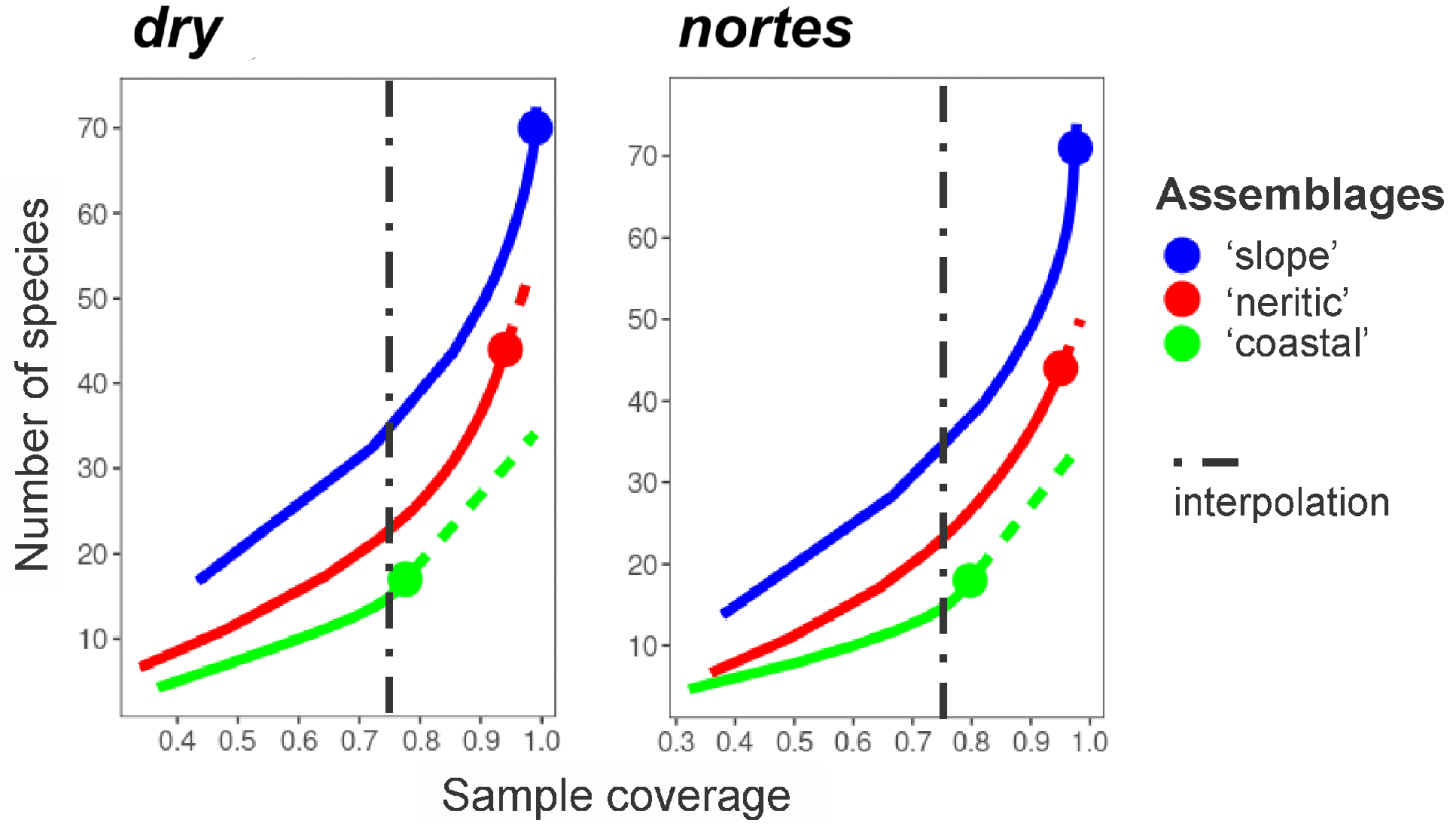
Estimation of diversity using the completeness method in the cross-shelf amphipod assemblages of the neritic province of the southern Gulf of Mexico, during *dry* and *nortes.*

Diversity showed an increasing pattern in a coastal-oceanic direction when comparing assemblages using the common completeness value of 0.75. During both seasons, 15 species were recorded in the ‘coastal’ assemblage, 23 in the ‘neritic’, and 35 in the ‘slope’ (Fig 6).

#### Vertical assemblages.

**Fig 7:**
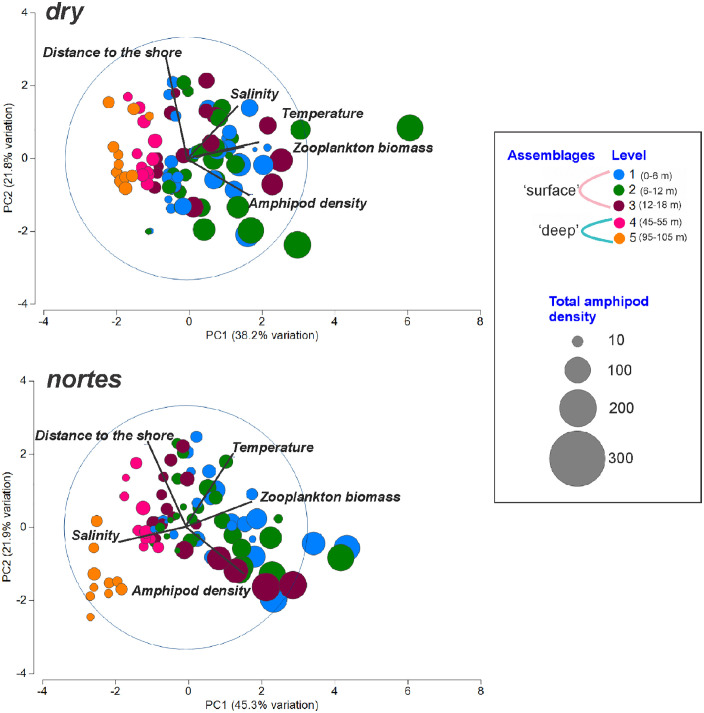
Representation of vertical assemblages resulting from the Principal Component Analysis (PCA) applied to data obtained during *dry* and *nortes.* Size of bubbles corresponds to the total amphipod density transformed into square root.

During *dry*, the PCA results indicated a clear separation between the ‘surface’ (levels 1–3: 0–18 m) and ‘deep’ (levels 4 and 5: 45–105 m) assemblages (Fig 7); the highest zooplankton biomass was associated with the levels that compose the ‘surface’ assemblage, which showed a higher amphipod density. During *nortes*, the ‘deep’ assemblage was more heterogeneous, since level 4 (45–55 m) was environmentally closer to the ‘surface’ assemblage, which was characterized by low salinity (Fig 7). Indeed, *R* values from the ANOSIM test confirmed the greater affinity of the level 4 (45–55 m depth) with the surface levels (0–18 m depth) during *nortes*, compared to *dry* (Table 3). Differences between the vertical assemblages (‘surface’ and ‘deep’) were confirmed by the ANOSIM test (*dry*: *R* = 0.411, *p* = 0.0001; *nortes*: *R* = 0.24, *p* = 0.0003). The complementary analysis excluding *L. bengalensis* showed no significant differences between the two assemblages in the vertical plane in both seasons (S1 Text: ANOSIM test, *p* > 0.05). The highest density of individuals (>1,000 ind/1,000 m^3^) was recorded in the ‘surface’ assemblage in both seasons (Figs 5 and 7).

**Table 3:** *R* values and significance (in brackets) resulting from the ANOSIM test applied to the zooplankton sampling levels, southern Gulf of Mexico. Upper matrix corresponds to the *dry*, and the lower one to *nortes*.

Levels	1: 0–6 m	2: 6–12 m	3: 12–18 m	4: 45–55 m	5: 95–105 m
1: 0–6 m	–	0.016 (0.204)	0.082 (0.062)	0.397 (0.0001)	0.568 (0.0001)
2: 6–12 m	−0.026 (0.89)	–	0.019 (0.30)	0.405 (0.0001)	0.618 (0.0001)
3: 12–18 m	0.005 (0.391)	0.011 (0.325)	–	0.319 (0.0007)	0.63 (0.0001)
4: 45–55 m	0.21 (0.01)	0.171 (0.015)	0.126 (0.038)	–	0.267 (0.0004)
5: 95–105 m	0.389 (0.0002)	0.305 (0.0008)	0.349 (0.0003)	0.121 (0.055)	–

Again, *L. bengalensis* contributed the most to the discrimination of the vertical assemblages, as shown in the SIMPER analysis, since it represents more than 20% of the dissimilarity between them during the studied periods (Table 4). In the supplementary analysis excluding *L. bengalensis,* the rest of the species differentiating the vertical assemblages (e.g., juveniles of *Primno, T. forcipatus*) (Table E in S1 Text) were the same as in the main analysis (Table 4).

**Table 4:** Species discriminating the vertical amphipod assemblages according to the SIMPER analysis during *dry* and *nortes.*

*Dry*		*Nortes*	
Taxa	Contribution %	Taxa	Contribution %
	‘surface’ vs ‘deep’ Av. Diss. = 79.29		‘surface’ vs ‘deep’ Av. Diss. = 74.61
*L. bengalensis*	20.20	*L. bengalensis*	24.44
*Primno* juveniles	5.54	*T. forcipatus*	4.94
*H. stephenseni*	4.20	Eupronoidae juv.	4.89
Eupronoidae juv.	3.87	*B. crusculum*	3.81
*A. blossevillei*	3.60	*Lycaea* sp.	3.13

**Fig 8:**
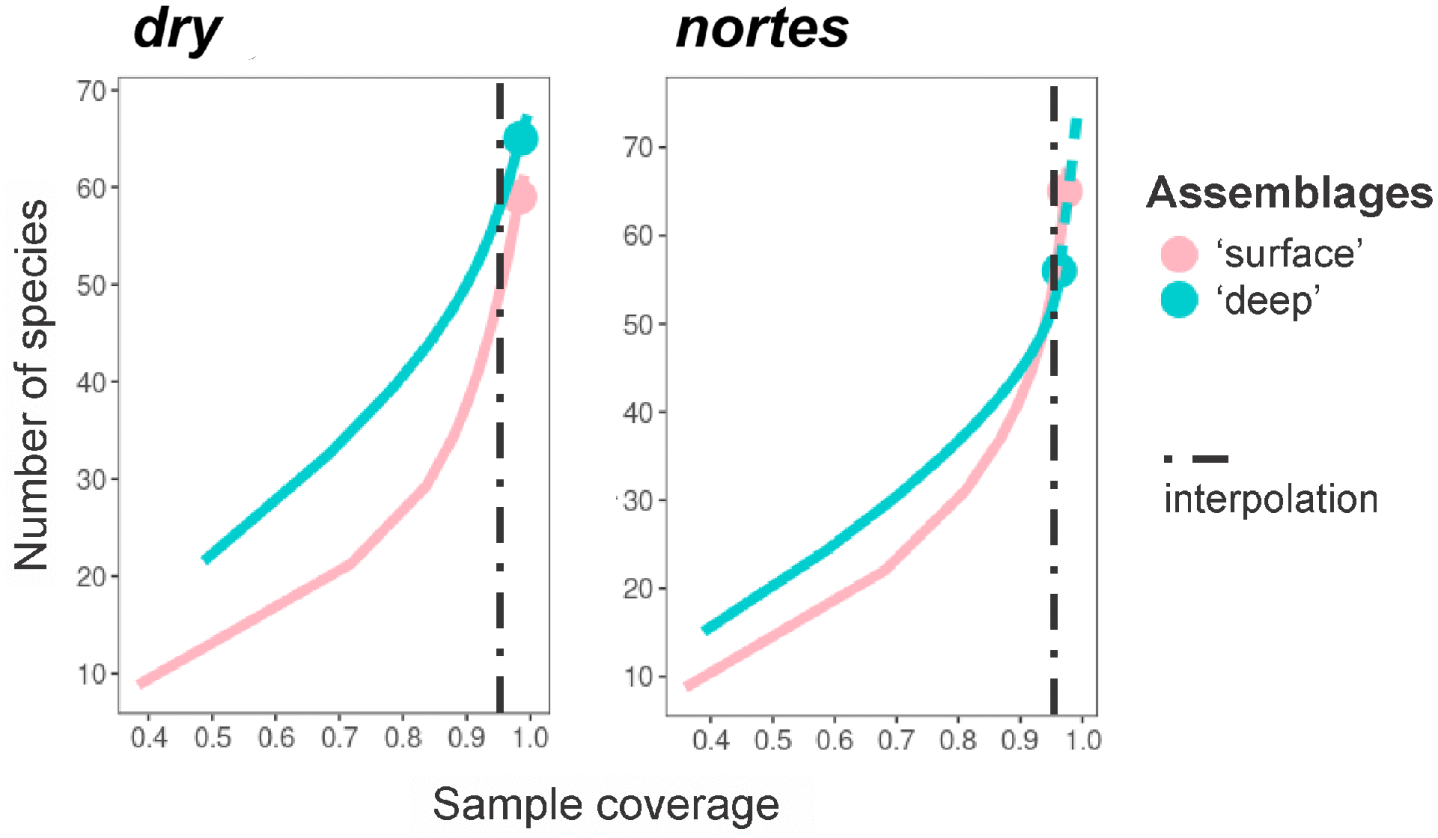
Estimation of diversity using the completeness method in the vertical amphipod assemblages of the neritic province of the southern Gulf of Mexico, during *dry* and *nortes.*

Diversity showed a clear stratification during the *dry*. When comparing the diversity with a common completeness value of 0.95, the ‘deep’ assemblage (58 species) was more diverse than the ‘surface’ one (50 species). In the *nortes*, no differences in diversity (55 species) were observed between assemblages (Fig 8).

## Discussion

### Seasonality

Worldwide, the ecology of pelagic amphipods in neritic waters has been poorly studied. The current study, conducted in the neritic province of the southern Gulf of Mexico, aimed to determine the effects of continental water discharges and wind regimes on the pelagic amphipod community. Seasonally, overall amphipod abundance showed significant differences (ANOSIM test, *p* < 0.05), with higher values during the *nortes* ($\bar{x}$ = 1,170.8 ± 2,098.9 ind/1,000 m^3^) compared to the *dry* ($\bar{x}$ = 1,059.5 ± 1,538.5 ind/1,000 m^3^). In the Ross River in Australia, nutrient discharges into the marine environment caused an increase in the abundance of hyperiid amphipods [14]. In the Gulf of Mexico, previous studies have indicated that at the beginning of *nortes,* a significant continental discharge occurs into the neritic province of the southern Gulf of Mexico [32,50,51], primarily due to the influence of the Grijalva-Usumacinta System (approximately 2,678 m^3^/s) [26]. Runoff greatly affects the amount of nutrients and sediments in the marine environment, leading to variations in the secondary production of surrounding waters that depend on the intensity of discharges throughout the year [51]. In this study, the higher correlation of the runoff (related to salinity) and productivity (related to zooplankton biomass) during *nortes* was evidenced in the PCA (Table 1). Enriching marine waters by continental discharges could positively affect the primary and secondary production at the beginning of the *nortes*, which can explain the observed differences in amphipod density.

### Cross-shelf assemblages

Results showed that differences among the horizontal assemblages were more evident during the *dry* (ANOSIM test, *p* < 0.05); during *nortes,* no significant differences were found between the ‘neritic’ and the ‘coastal’ assemblages (ANOSIM test, *p >* 0.05). This could be a consequence of the strong winds (8 m/s) characteristic of the *nortes* [34–36], which homogenize the hydrological conditions. Generally, the density of cross-shelf assemblages showed the following order: ‘neritic’, ‘coastal’, and ‘slope’ regardless of the season (S1 Table). In the *dry*, the PCA showed that zooplankton biomass was the variable with the greatest influence on the amphipod density (Table 1), which was higher in the ‘neritic’ assemblage (Fig 4). This is consistent with the previous observations in the oceanic province of the Gulf during the summer, since it is during this season when the zooplankton biomass increases as well as the density of amphipods [9], probably due to their affinity to inhabit the high productivity areas where they find their food [3,9,52]. During *nortes*, salinity was the most influential factor over the amphipods (Fig 4; Table 1). The lowest salinity zones in both seasons (Fig 3) coincided with the lowest amphipod density in the ‘coastal’ assemblage (Fig 5, Transect III), suggesting that pelagic amphipods avoid low-salinity waters derived from the continental discharges. Certainly, salinity conditions are a critical factor affecting the distribution of species because of the marine origin of amphipods and their low tolerance to low-salinity waters [1,15]. However, in addition to the continental discharges and the wind regime, other oceanographic processes can influence the zooplankton community in this area, such as marine currents [18–21], thermocline depth [22], and upwelling events [23] (among others), which also need further exploration.

*Lestrigonus bengalensis* was the species with the greatest contribution to the discrimination of assemblages in both seasons (Table 2), due to its high abundance (> 1,000 ind/1,000 m^3^) (S1 Table). The dominance of this species in the neritic province has been previously recorded in the Gulf of Mexico [3,17], but also in other tropical regions of the world [1,13,53,54]. This species has a broad tolerance to hydrological variability [14,15,53], and this was probably the reason why this species dominated in the horizontal assemblages, even in the lowest salinity zones (~30 psu) located in front of the Grijalva-Usumacinta System during *nortes*. This species registered the highest density values in the ‘neritic’ assemblage, following by the ‘coastal’, and decreased considerably in the ‘slope’ in both seasons (S1 Table); as stated, this could be a consequence of *L. bengalensis*’ preference for inhabiting neritic waters [1,13,17]. Due to its carnivorous habits, this species increases its density in high productivity areas where a higher food availability exists [14,52]. The relevance of this species in the neritic province as an indicator of the influence of hydro-meteorological variability on the horizontal plane was evidenced in a supplementary analysis excluding this species, which showed more homogeneity between the ‘coastal’ and the ‘neritic’ assemblages during both seasons (S1 Text). In the oceanic province, the dominance and relevance of this species as an indicator of the environmental variability could vary seasonally, since other taxa (e.g., juveniles of the *Primno* genus, *Anchylomera blossevillei*, and *Stenopleura atlantica*) may exceed the density of *L. bengalensis* during the summer [9,52].

The other three relevant species in the discrimination between assemblages were *A. blossevillei, T. forcipatus,* and the juveniles of Eupronoidae (Table 2). The ‘coastal’ assemblage was characterized by the higher density of *T. forcipatus* during both seasons, and the juveniles of Eupronoidae in *nortes* (S1 Table). The former taxon showed its highest abundance in the ‘coastal’ assemblage in both seasons (S1 Table). Indeed, the distribution pattern of this species shows a rare case among amphipods in which abundance decreases from the coast to the edge of the shelf, and whose hydrological tolerances are worth further study. For the juveniles of Eupronoidae, it seems that the hydrological conditions characterizing this assemblage during *nortes* represent a favorable environment for the reproduction of some members of the family, whose identity is unknown. In our study, we recorded seven Eupronoidae species –five from *Eupronoe* and two from *Parapronoe*– whose reproductive periods are unknown and may differ from one species to another throughout the year.

The ‘neritic’ assemblage was characterized by the high density of *A. blossevillei* and *T. forcipatus* (S1 Table) depending on the season. During *dry*, *A. blossevillei* was the most abundant species, but its density decreased drastically (approximately 45 times) in *nortes* (S1 Table). This species prefers to inhabit high-salinity waters [55], and this is probably the reason why the species’ density decreased in *nortes* and was not found in the ‘coastal’, where low-salinity waters occur (Fig 3). The little-known species *T. forcipatus* showed a clear preference for inhabiting the neritic waters during both seasons, but reached its highest density during *nortes* in the ‘neritic’ assemblage (S1 Table), suggesting its affinity to this season.

The ‘slope’ assemblage was characterized by the largest number of species and the high density of Eupronoidae juveniles, and *A. blossevillei* (S1 Table). In particular, the dominance of Eupronoidae juveniles in this assemblage is very interesting, since they also predominate in the ‘coastal’ assemblage. This could be a consequence of the fact that “Eupronoidae juveniles” may represent a group of different species whose hydrological preferences and tolerances are also different. Therefore, it is likely that the species responsible for the high density in the ‘slope’ assemblage were different from those that dominated in the ‘coastal’. Among the Eupronoidae species recorded in this work, *Eupronoe minuta* and *Eupronoe intermedia* showed their highest density in the ‘slope’ assemblage in both seasons (S1 Table), and these are probably the species responsible for this pattern. The preference of *E. minuta* and *E. intermedia* for inhabiting the oceanic environment is well known in the Gulf, especially *E. intermedia* [9,10,16].

Diversity increased from the coast to the ocean in both seasons (Fig 6). The increase in diversity towards the ‘slope’ is due to the oceanic nature of the pelagic amphipods [1]. In this study, some taxa from the infraorders Physocephalata (*Phrosinidae, Hyperioides, Tryphana,* most species of *Oxyscephalus, Phronima, Streetsia, Vibilia,* and *Paraphronima*), all Physosomata species, and the non-hyperiid *S. atlantica* were only found in the ‘slope’ assemblage (S1 Table), whose preferences for inhabiting the oceanic province have been widely recognized [1,8,9,56]. Seasonally, no differences in diversity were observed when comparing the corresponding horizontal assemblages (Fig 6), which contrasts with previous records in the oceanic province of the Gulf, where higher diversity has been recorded during the warm season compared to the cold one [9,10].

### Vertical assemblages

The highest density was observed in the ‘surface’ assemblage in both seasons (Figs 5 and 7), which coincides with other records of amphipods [54,57,58], and other zooplankton groups [28–31,59,60]. The high productivity of surface waters promotes an increase in the concentration of zooplankton organisms and, consequently, their trophic interactions in this area [61]. *Lestrigonus bengalensis* had the highest contribution in the vertical assemblages’ discrimination in the two seasons (Table 4). This species prefers to inhabit surface waters [1,54], where higher food availability exists. The dissimilarities between the two assemblages were greater during the *dry* than in *nortes*, because the environmental conditions of level 4 (45–55 m) during *nortes* could influence the vertical distribution of amphipods, as observed in other zooplankton groups [62]. Seasonal differences among vertical assemblages were only evident when the dominant species was included; without considering *L. bengalensis*, the vertical structure of the community was homogeneous regardless of season (S1 Text), highlighting the importance of this species in the neritic province.

Assemblages’ diversity varied seasonally. During the *dry,* the highest diversity was observed in the ‘deep’ assemblage. In contrast, diversity in both assemblages was more homogeneous during *nortes* (Fig 8), probably due to the effect of the more intense wind regime [34–36]. The above contrasts with the most recent study of pelagic amphipods in the oceanic province of the northwestern Caribbean Sea, since diversity was higher in the upper 50 m strata during the same season (*nortes*); this pattern was related to the day-night migration of the species [58]. However, the effect of the day-night vertical migration of species on diversity may vary between the neritic and the oceanic environments because the vertical migration range of pelagic amphipods is generally about 100 m [54], which cannot always be achieved in the neritic province.

## Conclusions

This study explored the horizontal and vertical variations in pelagic amphipod community structure in neritic waters of the southern Gulf of Mexico during two contrasting seasons: *dry* and *nortes*, differing in the volume of fluvial discharges and wind patterns. Results revealed that the greatest stratification of the cross-shelf and vertical assemblages occurred during the *dry*. In *nortes*, the effect of strong winds reduced the stratification: horizontally, there were no significant differences between the ‘neritic’ and ‘coastal’ assemblages; vertically, the assemblages were more homogeneous. The environmental variables with the greatest influence on the amphipod density were zooplankton biomass (*proxy* of food availability) and salinity, in response to the oceanic nature of these animals and their carnivorous habits. *Lestrigonus bengalensis* was dominant and represented a key species for detecting hydro-meteorological variation in the neritic province; without this species, the community showed a greater homogeneity regardless of the season. Horizontally, diversity increased from coast to ocean in both seasons, due to the amphipods’ oceanic preferences. Vertically, diversity was clearly different during *dry*, with higher diversity in the ‘deep’ assemblage; during *nortes*, diversity was the same in the two assemblages, which was related to the homogenizing effect of strong winds. Our results enrich the knowledge about the pelagic amphipod ecology and provide valuable information on the effects of the variability of wind regime and continental discharges on the amphipods.

## Supporting information

S1 Table Mean density (ind/1,000 m^3^) of the pelagic amphipods recorded in the neritic province of the southern Gulf of Mexico, during *dry* and *nortes* (1995). *n *= number of samples. (DOCX)

S1 Text Pelagic amphipod community structure without *Lestrigonus bengalensis.* (DOCX)
