# Supplementary material for: Cross-shelf and vertical structure of pelagic amphipods (Crustacea) related to hydro-meteorological conditions in the neritic zone, southern Gulf of Mexico
Source: PLoS One. 2025 Dec 4;20(12):e0336930. doi: 10.1371/journal.pone.0336930 (PMC12677503; doi:10.1371/journal.pone.0336930)
Supplement: S1 Table — (DOCX) [file pone.0336930.s001.docx]

**S1 Table. Mean density (ind/1,000 m^3^) of the pelagic amphipods recorded in the neritic province of the southern Gulf of Mexico, during *dry* and *nortes* (1995).** *n* = number of samples.

|  | *Dry* | | | *Nortes* | | |
| --- | --- | --- | --- | --- | --- | --- |
| Taxa | ‘coastal’ | ‘neritic’ | ‘slope’ | ‘coastal’ | ‘neritic’ | ‘slope’ |
|  | (*n*= 9) | (*n*= 34) | (*n*= 50) | (*n*= 9) | (*n*= 34) | (*n*= 50) |
| **Suborder Amphilochidea** |  |  |  |  |  |  |
| **Family Eurytheneidae** |  |  |  |  |  |  |
| *Eurythenes* sp. | - | 0.34 | 0.25 | - | 1.61 | 0.18 |
| **Family Synopioidea** |  |  |  |  |  |  |
| *Synopia ultramarina* | - | 1.66 | 0.42 | - | 0.26 | - |
|  |  |  |  |  |  |  |
| **Suborder Senticaudata** |  |  |  |  |  |  |
| **Family Corophiidae** |  |  |  |  |  |  |
| Corophiidae sp. | 1.23 | 0.28 | 0.1 | - | 0.09 | - |
| **Family Corophiidae** |  |  |  |  |  |  |
| *Stenopleura atlantica* | - | - | 5.57 | - | - | 1.56 |
|  |  |  |  |  |  |  |
| Unidentified non-hyperiids | 5.83 | 0.91 | - | - | 0.43 | 0.1 |
|  |  |  |  |  |  |  |
| **Suborder Hyperiidea** |  |  |  |  |  |  |
| **Infraorder Phyosocephalata** |  |  |  |  |  |  |
| **Superfamily Phronimoidea** |  |  |  |  |  |  |
| **Family Lestrigonidae** |  |  |  |  |  |  |
| *Hyperietta luzoni* | - | - | - | 0.52 | - | 1.18 |
| *Hyperietta stebbingi* | - | - | - | - | - | 0.1 |
| *Hyperietta stephenseni* | 0.51 | 3.2 | 14.76 | 1.03 | 0.68 | 2.8 |
| *Hyperietta vosseleri* | 1.2 | 1.95 | 8.79 | 5.68 | 7.98 | 4.23 |
| *Hyperioides longipes* | - | - | 3.69 | - | - | 1.01 |
| *Hyperioides sibaginis* | - | - | - | - | - | 0.21 |
| *Hyperionyx macrodactylus* | - | - | 0.41 | - | 0.4 | 1.91 |
| *Lestrigonus bengalensis* | 1,024.78 | 1,800.9 | 201.14 | 1,018.48 | 1,527.12 | 642.66 |
| *Lestrigonus latissimus* | - | - | 0.79 | - | - | 0.2 |
| *Lestrigonus macrophthalmus* | 5.92 | 2.22 | 9.75 | - | 0.51 | 3.56 |
| *Lestrigonus schizogeneios* | - | 1.14 | 3.98 | - | - | 0.4 |
| *Lestrigonus shoemakeri* | - | 0.28 | 2.63 | 1.2 | 1.3 | 4.97 |
| *Phronimopsis spinifera* | - | 0.31 | 2.97 | - | 0.08 | 0.78 |
| *Themistella fusca* | 5.35 | 8.08 | 8.07 | 17.65 | 10.54 | 7.09 |
| Lestrigonidae juveniles/unidentified | - | - | - | 10.15 | 8.5 | 1.29 |
| **Family Phronimidae** |  |  |  |  |  |  |
| *Phronima atlantica* | - | - | 2.2 | - | 1.69 | 3.94 |
| *Phronima colletti* | - | - | 0.23 | - | 0.66 | 1.07 |
| *Phronima curvipes* | - | 0.22 | 0.05 | - | - | - |
| *Phronima pacifica* | - | 1.95 | 13.69 | - | 1.26 | 3.54 |
| *Phronima sedentaria* | - | - | 0.22 | - | 0.08 | 0.07 |
| *Phronima solitaria* | - | - | - | - | 0.15 | - |
| *Phronima stebbingi* | - | - | 0.05 | - | 0.33 | 0.14 |
| *Phronimella elongata* | - | 0.24 | 2.18 | - | - | 0.07 |
| **Family Phrosinidae** |  |  |  |  |  |  |
| *Anchylomera blossevillei* | - | 27.14 | 30.07 | - | 0.6 | 1.64 |
| *Phrosina semilunata* | - | - | 0.94 | - | - | 0.46 |
| *Primno abyssalis* | - | - | - | - | - | 0.36 |
| *Primno brevidens* | - | - | 1.39 | - | - | 0.86 |
| *Primno evansi* | 0.24 | 0.71 | 6.22 | - | - | 0.37 |
| *Primno latreillei* | - | 1.01 | 7.06 | - | - | 0.4 |
| *Primno* juveniles | - | 0.13 | 20.17 | - | - | - |
|  |  |  |  |  |  |  |
| **Superfamily Platysceloidea** |  |  |  |  |  |  |
| **Family Amphithyridae** |  |  |  |  |  |  |
| *Amphithyrus bispinosus* | - | - | 0.09 | - | - | 0.06 |
| *Amphithyrus muratus* | - | 0.17 | 1.23 | - | - | - |
| *Amphithyrus sculpturatus* | - | - | 0.76 | - | 0.08 | 0.91 |
| *Paralycaea gracilis* | - | - | - | - | - | 0.31 |
| **Family Brachyscelidae** |  |  |  |  |  |  |
| *Brachyscelus crusculum* | - | 1.73 | 2.22 | 17.63 | 23.37 | 11.68 |
| *Brachyscelus globiceps* | 0.66 | 15.07 | 16.29 | 9.53 | 0.35 | 2.5 |
| *Brachyscelus rapacoides* | - | - | - | - | - | 0.1 |
| *Brachyscelus rapax* | - | - | - | - | - | 0.1 |
| **Family Eupronoidae** |  |  |  |  |  |  |
| Eupronoidae juveniles | 2.11 | 15.92 | 45.15 | 32.68 | 18.72 | 28.8 |
| *Eupronoe armata* | *-* | *-* | *-* | - | - | 0.08 |
| *Eupronoe intermedia* | 0.66 | 16.3 | 18.98 | 0.52 | 6.04 | 8.47 |
| *Eupronoe laticarpa* | - | 0.13 | - | - | 0.3 | 0.49 |
| *Eupronoe maculata* | - | - | 2.02 | - | 0.11 | 0.99 |
| *Eupronoe minuta* | - | 0.67 | 1.35 | - | 0.26 | 1.77 |
| *Parapronoe crustulum* | - | - | 0.31 | - | - | - |
| *Parapronoe parva* | - | 0.13 | 0.18 | - | - | 1.08 |
| **Family Lycaeidae** |  |  |  |  |  |  |
| *Lycaea* sp. | 1.86 | 6.95 | 15.89 | 0.52 | 6.25 | 13.82 |
| *Simorhynchotus antennarius* | 1.01 | 1.98 | 3.48 | 5.4 | 4.89 | 4.37 |
| **Family Lycaeopsidae** |  |  |  |  |  |  |
| *Lycaeopsis themistoides* | - | 4.24 | 4.22 | - | 1.08 | 5.69 |
| *Lycaeopsis zamboangae* | - | 0.51 | 2.97 | 1.03 | 0.41 | 4.13 |
| **Family Oxycephalidae** |  |  |  |  |  |  |
| *Cranocephalus scleroticus* | 1.32 | 10.39 | 4.39 | *-* | *-* | *-* |
| *Leptocotis tenuirostris* | - | 0.2 | 1.46 | - | 0.15 | 0.75 |
| *Oxycephalus clausi* | - | - | - | - | 0.1 | 1.15 |
| *Oxycephalus latirostris* | - | - | 0.05 | - | - | - |
| *Oxycephalus piscator* | - | - | 1.32 | 0.4 | - | 0.17 |
| *Rhabdosoma whitei* | - | 0.5 | 0.55 | - | 0.29 | 0.14 |
| *Streetsia challengeri* | - | 0.13 | 0.23 | - | - | 0.07 |
| *Streetsia porcella* | - | - | 0.15 | - | - | 0.27 |
| *Streetsia steenstrupi* | - | - | - | - | - | 0.08 |
| **Family Parascelidae** |  |  |  |  |  |  |
| *Parascelus edwardsi* | 0.51 | 1.33 | 0.91 | 0.52 | 0.54 | 0.38 |
| *Schizoscelus ornatus* | - | 0.2 | 0.14 | - | - | - |
| *Thyropus sphaeroma* | - | 1.38 | 1.5 | 1.2 | 1.83 | 4.86 |
| **Family Platyscelidae** |  |  |  |  |  |  |
| *Hemityphis tenuimanus* | - | 0.1 | 2.89 | - | 0.48 | 0.32 |
| *Paratyphis promontori* | 0.7 | 4.79 | 12.38 | - | 1.46 | 3.27 |
| *Platyscelus armatus* | - | 0.13 | 2.1 | - | - | - |
| *Platyscelus crustulatus* | - | 0.9 | 0.33 | - | 0.08 | 1.47 |
| *Platyscelus serratulus* | - | - | 0.71 | - | - | - |
| *Tetrathyrus forcipatus* | 19.33 | 17.59 | 14.59 | 34.79 | 43.82 | 18.4 |
| **Family Tryphanidae** |  |  |  |  |  |  |
| *Tryphana malmi* | - | - | 0.64 | - | - | - |
|  |  |  |  |  |  |  |
| **Superfamily Vibiloidea** |  |  |  |  |  |  |
| **Family Paraphronimidae** |  |  |  |  |  |  |
| *Paraphronima crassipes* | - | - | 0.36 | - | - | 0.64 |
| *Paraphronima gracilis* | - | - | 1.56 | - | 0.08 | 0.28 |
| **Family Vibilidae** |  |  |  |  |  |  |
| *Vibilia australis* | - | - | 0.24 | - | - | - |
| *Vibilia chuni* | *-* | - | - | - | - | 0.1 |
| *Vibilia propinqua* | - | - | 0.16 | - | - | 0.07 |
| *Vibilia stebbingi* | - | 0.47 | 0.78 | - | 1.43 | 2.07 |
| *Vibilia viatrix* | - | - | 0.12 | - | 0.42 | 1.88 |
|  |  |  |  |  |  |  |
| **Infraorder Physosomata** |  |  |  |  |  |  |
| **Family Scinidae** |  |  |  |  |  |  |
| *Acanthoscina acanthodes* | - | - | 0.05 | - | - | - |
| *Scina borealis* | - | - | 0.17 | - | - | 0.76 |
| *Scina crassicornis* | - | - | 0.16 | - | - | 0.62 |
| *Scina nana* | - | - | 0.18 | - | - | - |
| *Scina typhlops* | - | - | - | - | - | 0.08 |
|  |  |  |  |  |  |  |
| **Overall mean density** | 1,073.2 | 1,954.6 | 511.1 | 1,158.9 | 1,676.8 | 811.6 |
