# Supplementary material for: Cross-shelf and vertical structure of pelagic amphipods (Crustacea) related to hydro-meteorological conditions in the neritic zone, southern Gulf of Mexico
Source: PLoS One. 2025 Dec 4;20(12):e0336930. doi: 10.1371/journal.pone.0336930 (PMC12677503; doi:10.1371/journal.pone.0336930)
Supplement: S1 Text — (DOCX) [file pone.0336930.s002.docx]

**S1 Text. Pelagic amphipod community structure without *Lestrigonus bengalensis***

In this analysis, without considering *Lestrigonus bengalensis,* amphipod density was higher during the *dry* season, with 229.1 ± 277.4 ind/1,000 m^3^, while in *nortes* it was 158.9 ± 170.6 ind/1,000 m^3^. The ANOSIM test revealed significant differences in amphipod density between seasons (*R*= 0.104, *p* = 0.0001), as in the complete analysis, that is, when *L. bengalensis* was included.

In comparison with the complete analysis, a greater homogeneity in the community structure was observed in this analysis, since no significant differences were detected between the horizontal assemblages ‘neritic’ and ‘coastal’ (ANOSIM test; *dry* *p* = 0.153, *nortes* *p* = 0.328), neither between the vertical ‘surface’ and ‘deep’ (ANOSIM test; *dry* *p* = 0.176, *nortes* *p* = 0.189) in both seasons. This greater homogeneity in the community structure was evident in the representation of the first two axes of the PCA (Figures A and B). The PCA demonstrated that the variables with the greatest influence in the community coincided with those of the complete analysis: zooplankton biomass during *dry* and zooplankton biomass and salinity during *nortes* (Table C).

The SIMPER analysis showed that the species with the greatest contribution to the differentiation of the horizontal assemblages were similar to the complete analysis: juveniles of Eupronoidae, *Tetrathyrus forcipatus*, and *Anchylomera blossevillei* (Table D). The same occurred in the vertical plane, where juveniles of the *Primno* genus stand out in *dry* and *T. forcipatus* in *nortes* (Table E).

In summary, the main differences between this and the complete one lie in the greater homogeneity of the amphipod community in both the horizontal and the vertical planes, regardless of the hydro-meteorological conditions that characterize each season. From the above, we deduce the importance of *L. bengalensis* in the amphipod community structure in the horizontal and vertical planes in the neritic province of the southern Gulf.

**
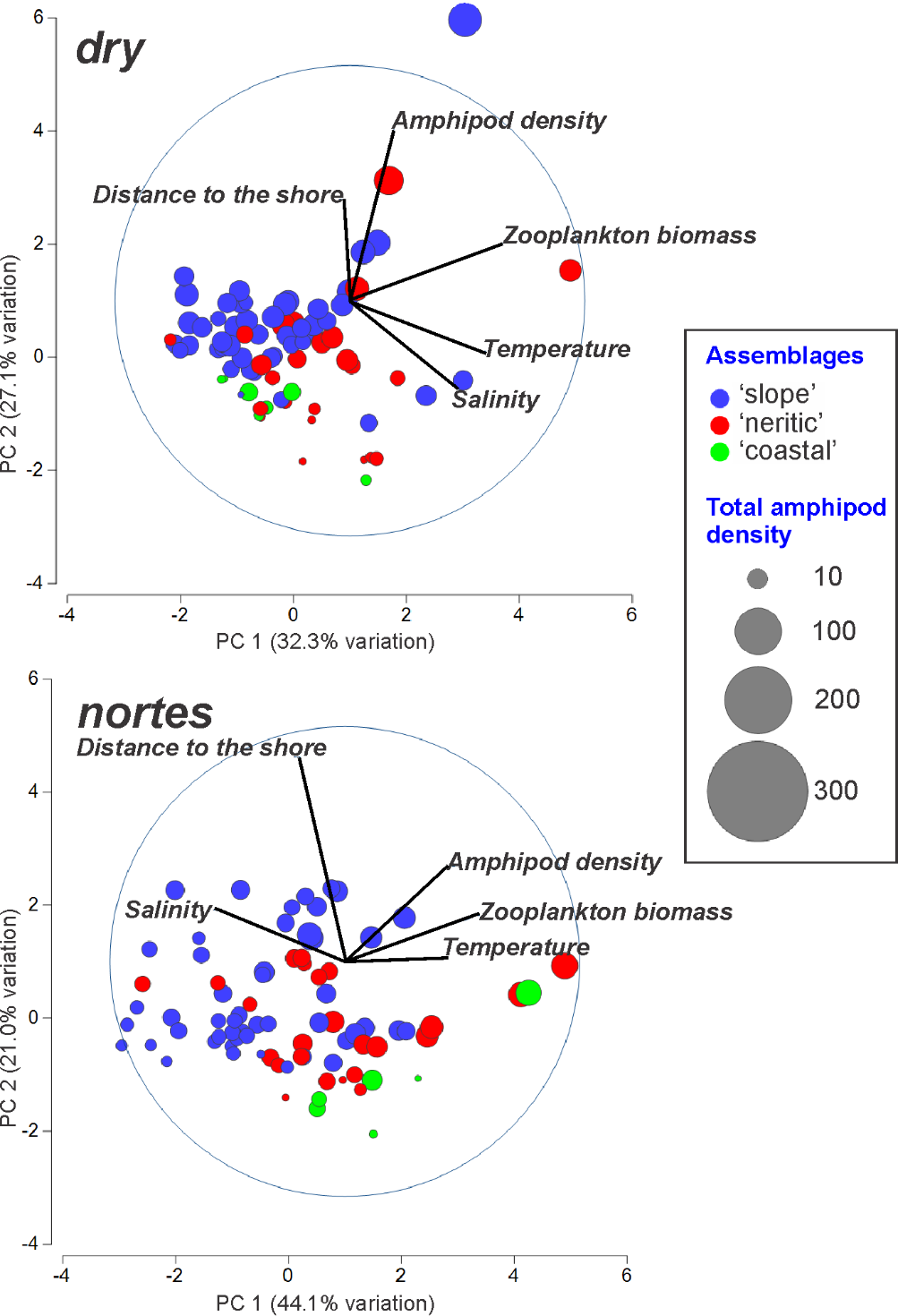
**

**Fig A. Representation of horizontal assemblages resulting from the Principal Component Analysis (PCA) applied to data obtained during *dry* and *nortes*, excluding *Lestrigonus bengalensis*.** Size of bubbles corresponds to the total amphipod density transformed into square root.


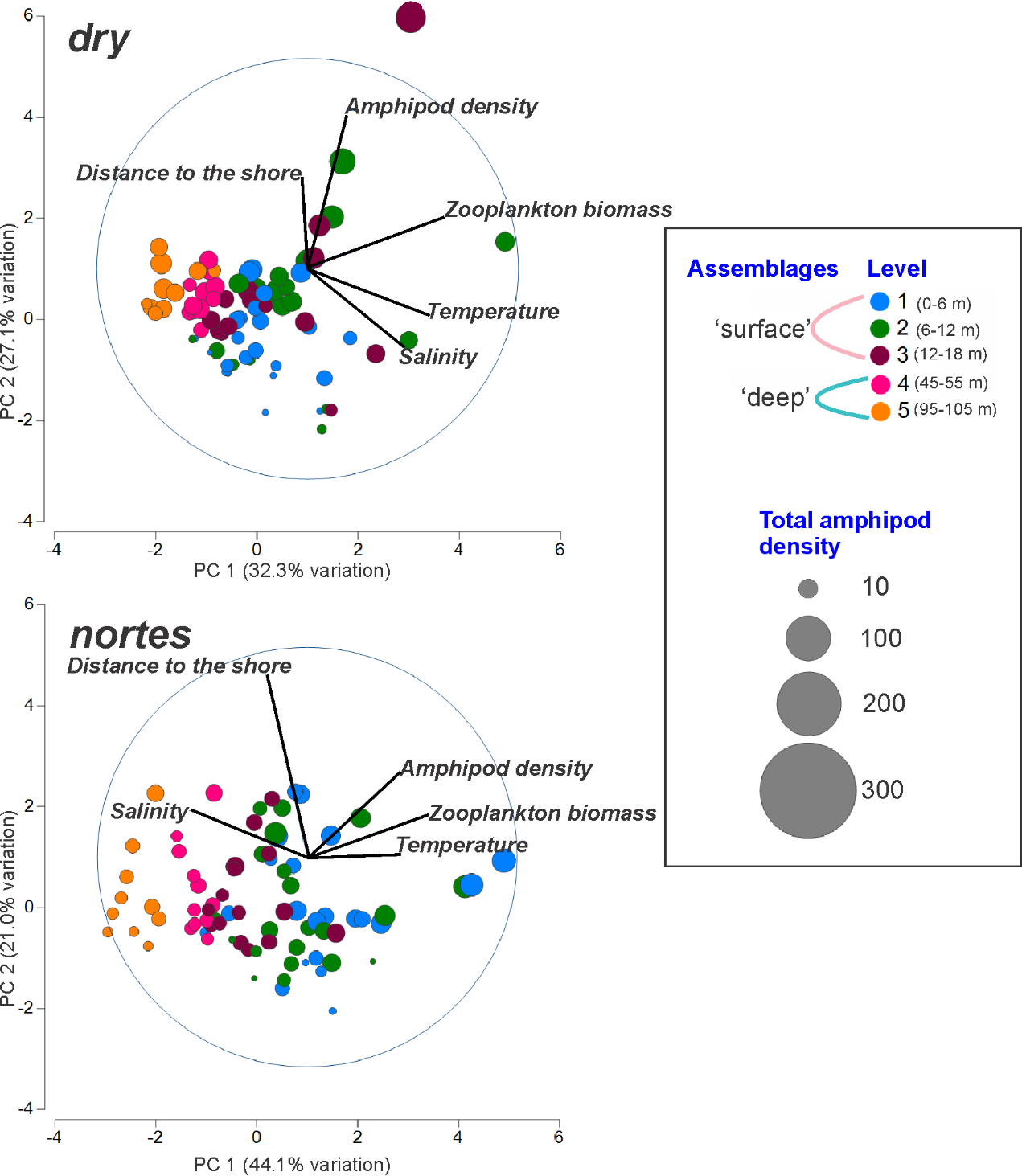


**Fig B.** **Representation of vertical assemblages resulting from the Principal Component Analysis (PCA) applied to data obtained during *dry* and *nortes*, excluding *Lestrigonus bengalensis*.** Size of bubbles corresponds to the total amphipod density transformed into square root.

|  | *Dry* | | *Nortes* | |
| --- | --- | --- | --- | --- |
| Variable | PC1 | PC2 | PC1 | PC2 |
| Distance to the shore | 0.022 | -0.428 | -0.193 | -0.015 |
| Temperature | -0.561 | 0.213 | 0.405 | -0.015 |
| **Salinity** | -0.467 | 0.382 | **-0.551** | -0.221 |
| **Zooplankton biomass** | **-0.655** | -0.247 | **0.553** | -0.192 |
| Amphipod density | -0.193 | -0.752 | 0.436 | -0.405 |
| Total variance (%) | 32.3 | 27.1 | 44.1 | 21.0 |

**Table C. Correlation between the variables and the first two axes of the Principal Component Analysis (without *Lestrigonus bengalensis*).**

**Table D.** **Species discriminating the cross-shelf amphipod assemblages (without *Lestrigonus bengalensis*) according to the SIMPER analysis during *dry* and *nortes*.**

| *Dry* | | *Nortes* | |
| --- | --- | --- | --- |
| Taxa | Contribution % | Taxa | Contribution % |
|  | ‘slope’ vs ‘neritic’  Av. Diss. = 84.45 |  | ‘slope’ vs ‘neritic’  Av. Diss. = 83.26 |
| Eupronoidae juv. | 7.69 | *T. forcipatus* | 9.08 |
| *A. blossevillei* | 7.45 | Eupronoidae juv. | 7.83 |
| *T. forcipatus* | 5.68 | *B. crusculum* | 6.42 |
|  | ‘slope’ vs ‘coastal’  Av. Diss. = 90.89 |  | ‘slope’ vs ‘coastal’  Av. Diss. = 86.71 |
| Eupronoidae juv. | 7.64 | Eupronoidae juv. | 8.74 |
| *A. blossevillei* | 7.38 | *T. forcipatus* | 8.06 |
| *T. forcipatus* | 5.93 | *B. crusculum* | 6.58 |
|  | ‘neritic’ vs ‘coastal’  Av. Diss. = 91.03 |  | ‘neritic’ vs ‘coastal’  Av. Diss. = 86.68 |
| *T. forcipatus* | 13.87 | *T. forcipatus* | 19.67 |
| Corophiidae | 9.02 | *S, antennarius* | 12.25 |
| Non-Hyperiids | 8.36 | *B. crusculum* | 10.43 |

**Table E. Species discriminating the vertical amphipod assemblages (without *Lestrigonus bengalensis*) according to the SIMPER analysis during *dry* and *nortes.***

| *Dry* | | *Nortes* | |
| --- | --- | --- | --- |
| Taxa | Contribution % | Taxa | Contribution % |
|  | ‘surface’ vs ‘deep’  Av. Diss. = 83.99 |  | ‘surface’ vs ‘deep’  Av. Diss. = 82.43 |
| *Primno* juveniles | 6.90 | *T. forcipatus* | 7.33 |
| *H. stephenseni* | 5.30 | Eupronoidae juv. | 6.29 |
| Eupronoidae juv. | 4.78 | *B. crusculum* | 5.38 |
| *A. blossevillei* | 4.30 | *T. fusca* | 4.33 |
